# Identifying ribosome heterogeneity using ribosome profiling

**DOI:** 10.1093/nar/gkac484

**Published:** 2022-06-10

**Authors:** Ferhat Alkan, Oscar G Wilkins, Santiago Hernández-Pérez, Sofia Ramalho, Joana Silva, Jernej Ule, William J Faller

**Affiliations:** Division of Oncogenomics, The Netherlands Cancer Institute, Amsterdam, The Netherlands; The Francis Crick Institute, London, UK; UCL Queen Square Motor Neuron Disease Centre, Department of Neuromuscular Diseases, UCL Queen Square Institute of Neurology, UCL, London, UK; Division of Oncogenomics, The Netherlands Cancer Institute, Amsterdam, The Netherlands; Division of Oncogenomics, The Netherlands Cancer Institute, Amsterdam, The Netherlands; Division of Oncogenomics, The Netherlands Cancer Institute, Amsterdam, The Netherlands; The Francis Crick Institute, London, UK; UCL Queen Square Motor Neuron Disease Centre, Department of Neuromuscular Diseases, UCL Queen Square Institute of Neurology, UCL, London, UK; UK Dementia Research Institute Centre, King’s College London, London, UK; Division of Oncogenomics, The Netherlands Cancer Institute, Amsterdam, The Netherlands

## Abstract

Recent studies have revealed multiple mechanisms that can lead to heterogeneity in ribosomal composition. This heterogeneity can lead to preferential translation of specific panels of mRNAs, and is defined in large part by the ribosomal protein (RP) content, amongst other things. However, it is currently unknown to what extent ribosomal composition is heterogeneous across tissues, which is compounded by a lack of tools available to study it. Here we present dripARF, a method for detecting differential RP incorporation into the ribosome using Ribosome Profiling (Ribo-seq) data. We combine the ‘waste’ rRNA fragment data generated in Ribo-seq with the known 3D structure of the human ribosome to predict differences in the composition of ribosomes in the material being studied. We have validated this approach using publicly available data, and have revealed a potential role for eS25/RPS25 in development. Our results indicate that ribosome heterogeneity can be detected in Ribo-seq data, providing a new method to study this phenomenon. Furthermore, with dripARF, previously published Ribo-seq data provides a wealth of new information, allowing the identification of RPs of interest in many disease and normal contexts. dripARF is available as part of the ARF R package and can be accessed through https://github.com/fallerlab/ARF.

## INTRODUCTION

Our understanding of ribosome composition has developed significantly in recent years. Ribosomes have long been considered a homogenous population, passively translating mRNA into protein, however this view has been challenged by a number of findings (reviewed in (1)). It is now known that there is substantial diversity in the ribosomal proteins (RPs), rRNA sequence and modifications, RP paralogues, ribosome-associated proteins and post translational modifications of RPs in the ribosome (2–7). This heterogeneity is known to regulate many biological processes as diverse as development, immunosurveillance, metabolism, cell cycle, and stress response, primarily through the preferential translation of specific panels of mRNAs (3,8–11). It has been shown, for example, that mRNAs bound to uL1/RPL10A containing ribosomes are far less efficiently translated by ribosomes not containing this RP (2), and that eL6/RPL6 and eL28/RPL28 play opposing roles in immunosurveillence (8). However, this field is still in its infancy, and the extent of this mechanism of regulation is currently unknown. This is compounded by a lack of techniques available to study ribosome heterogeneity. Mass spectrometry (MS) techniques are required to identify differential RP incorporation into the ribosome, and these are challenging due to the small size of RPs, and their lack of tryptic peptides. As a result, alternative methods are needed.

Ribosome Profiling (Ribo-seq) has become a cornerstone in the study of translational regulation. It is extensively used, and provides a huge amount of information on ribosome binding to mRNA. However, high levels of rRNA contamination are a common problem in this protocol, reducing the sequencing depth for protein-coding RNAs. As a result, rRNA reads are treated as contaminants, and every effort is made to experimentally reduce their abundance, although the efficiency of such depletion is very low (12) and known to introduce different biases (13). Sequenced rRNA reads that survive experimental depletion are often discarded in the first steps of the bioinformatic analysis of the data. However, there is the potential that rRNA fragment data holds valuable insights, particularly into the structure of the ribosome. As the presence or absence of RPs may change the degradation of rRNA during the RNase treatment step of Ribo-seq, there may be predictable changes in rRNA protection patterns that can be detected in the rRNA fragments sequenced.

Here, we present a new bioinformatic tool that integrates Ribo-seq produced rRNA fragment data with the 3D structure of the ribosome to predict changes in ribosome heterogeneity across samples. This is done by defining rRNA–RP contact points and comparing the relative abundance of the rRNA around these points in different conditions. Using this information, our Differential RP Incorporation Prediction by Analysis of rRNA Fragments (dripARF) tool predicts which RPs are differentially incorporated into the ribosome, making them prime candidates for differential ribosome heterogeneity across conditions.

We have validated this approach using publicly available experimental data, which shows that alterations of an RP in the ribosomes causes a detectable and predictable change in Ribo-seq produced rRNA fragments. We have also used it to probe differential RP incorporation in development, and identified a potential functional consequence of such heterogeneity.

Our novel bioinformatic approach allows significant additional information to be gleaned from Ribo-seq experiments, and provides a new much-needed tool to identify differential ribosome heterogeneity across conditions. dripARF is released as part of the *ARF* R package that is freely accessible at https://github.com/fallerlab/ARF.

## MATERIALS AND METHODS

### rRNA sequences

Four distinct ribosomal RNAs exist within the eukaryotic ribosome (28S, 18S, 5-8S, 5S), however, different species have differing numbers of copies of them, which sometimes also differ in sequence. For example, in humans there are five annotated sequence variants for 28S, 18S and 5-8S rRNAs. In this study, for simplification purposes, we have selected one variant for each rRNA. For human, our selection included the rRNAs with the following identifiers; NR_003287.4 (RNA28SN5), NR_003286.4 (RNA18SN5), NR_003285.3 (RNA5-8SN5) and A7 (4v6X (14)). For mouse, rRNAs with NR_003279.1 (Rn28s1), NR_003278.3 (Rn18s), NR_003280.2 (Rs5-8s1) and NR_030686.1 (Rn5s) accession ids were selected.

### Analysis of the ribosome 3D structure

The human ribosomal structure was downloaded as a CIF file from the Protein Data Bank (code 4V6X (14)). The CIF file was parsed using Bio3D (15). For each rRNA residue, the minimum distance to all 82 ribosomal proteins was calculated in angstroms (Å), using the N1 atom and alpha carbon atoms as references for RNA and protein respectively. Results were validated manually by direct visualisation of the structure in Pymol; it was also verified that the human rRNA sequences used during mapping exactly matched the sequences in the structural data. We created the human-specific rRNA-RP proximity matrix using the calculated RP-distances, and based on rRNA alignments between human and mouse, we created the mouse rRNA-RP proximity matrix in the following way. RP-distance value of every human rRNA residue is directly matched to its aligned mouse residue based on the sequence alignment. RP-distance values of mouse residues that are not aligned to any human residue (insertion in mouse rRNA when compared to human) are interpolated linearly based on RP-distances of the closest aligned residues.

### dripARF method

We have developed the ARF R package for the **A**nalysis of **R**ibosomal RNA **F**ragments that originate from Ribo-seq experiments. In this manuscript, we present the dripARF method that can detect which RPs give rise to differential ribosome heterogeneity across Ribo-seq produced samples.

The method is composed of four steps: identification of rRNA fragments, quantification of positional rRNA fragment abundance differences, extraction of RP–rRNA contact point sets and enrichment tests for the prediction of RPs. Due to potential differences in sequencing protocols, the first step of the method is handled by the user with a simple read-alignment of Ribo-seq produced reads to the rRNA sequences provided within the ARF package. In this manuscript, we have performed this alignment using the TopHat (v2.1.1) aligner (16), with the following parameter settings; *-n 2 --no-novel-juncs --no-novel-indels --no-coverage-search --segment-length 25*.

Positional quantification of rRNA fragments (Step 2) is the first step handled by the R package. With the user-given alignment files (.bam) or nt-level read coverage files (.bedGraph), the pipeline first quantifies how many rRNA fragments map to each nucleotide position on the reference rRNA sequences, using the HelloRanges package. After excluding positions with low number of rRNA fragments, positional abundances are normalized across samples and differential abundance analysis is performed, using the DESeq2 package (17). This step results in position specific log FC and *P*-values representing the rRNA fragment change between conditions. Step 3 of the pipeline is rather a static one, since it is not run repeatedly. In this step, based on the species-specific RP–rRNA proximity matrix, we create a RP contact set for every RP, representing the rRNA residues that are in close proximity to that RP. We defined a residue as being in contact with an RP if it was in the 5% of residues with the lowest rRNA-RP distance. By this definition, any residue within ∼27.4 Å of an RP is counted as a contact point. To avoid overcrowding of these sets, we also limited the set sizes to 360 contact points, prioritizing the residues in closest proximity to the RP. This threshold was chosen as it represents 5% of the total rRNA length. Additionally, in this step, for every RP contact set, we also create 100 corresponding background sets to use in the enrichment analysis. Background sets are simply created by shifting the contact residues by a certain distance at each turn, for which rRNAs are concatenated and assumed to be circular. This shift distance (71 for human and 67 for mouse) is decided based on the complete rRNA length of species, and corresponds to roughly 1% of total length.

Afterwards, in Step 4, we perform three enrichment analyses to test if changes in rRNA fragments are significantly biased towards certain RP contact sets. The first analysis is RP contact Set Enrichment Analysis (RPSEA) which is identical to Gene Set Enrichment Analysis (GSEA) (18) that is generally performed after differential expression analysis of genes. In RPSEA, rRNA positions and RP contact sets (and their corresponding background sets) are treated as genes and gene sets, respectively. In dripARF, this test is performed by the GSEA function from clusterProfiler R package (19), where the value abs(log FC) × max(−log_10_(adj *P*), 5) is used for ranking of rRNA positions. Based on this enrichment test we create two enrichment scores for every RP. Enrichment Score 1 directly corresponds to the normalized enrichment score (NES) of every RP contact set, however, Enrichment Score 2 (RPSEA_rand) represents its deviation from the enrichment of background sets. This is calculated by transforming the NES score of RP contact set to a *z*-score, based on the distribution of NES scores of its corresponding background sets. It is expected that ES1 and ES2 values correlate, however, ES2 enables comparison of ES1 across different RPs and to set a threshold for the selection of top candidate RPs. This threshold is set to 1 at default settings. Creation of ES2 can also be considered as an attempt for correcting the bias introduced by set size differences. Lastly, we also perform an overrepresentation analysis (ORA) as Enrichment Test 3 where we test if rRNA positions that have significant rRNA fragment abundance change (adj *P*< 0.05 & abs(log_2_FC) > 0.5) are overrepresented in certain RP contact sets. This is done using the fora function from fsgea R package which performs an hypergeometric test.

### Public datasets

**Figure 1.**
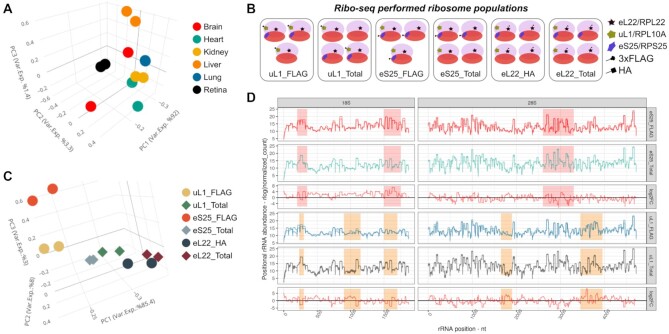
Sample specificity of Ribo-seq produced rRNA fragments. (**A**) PCA with position-specific rRNA abundance measures (regularized log of normalized count) shows that Ribo-seq produced rRNA fragments are consistently different across fetal tissues. (**B**) Schematic representation of different ribosome populations that are subjected to Ribo-seq in (2). (**C**) PCA with rRNA fragments from different ribosome populations shows consistent and clear separation of samples. (**D**) Comparison of position-specific rRNA abundances between selected populations shows the consistency across replicates (*n* = 2, separate lines in rows 1, 2, 4 and 5) and reveals that hotspots (highlighted) for rRNA fragment heterogeneity change across comparisons. Note that rows 3 and 6 indicate the differences between populations in rows 1, 2 and 4, 5, respectively.

**Figure 2.**
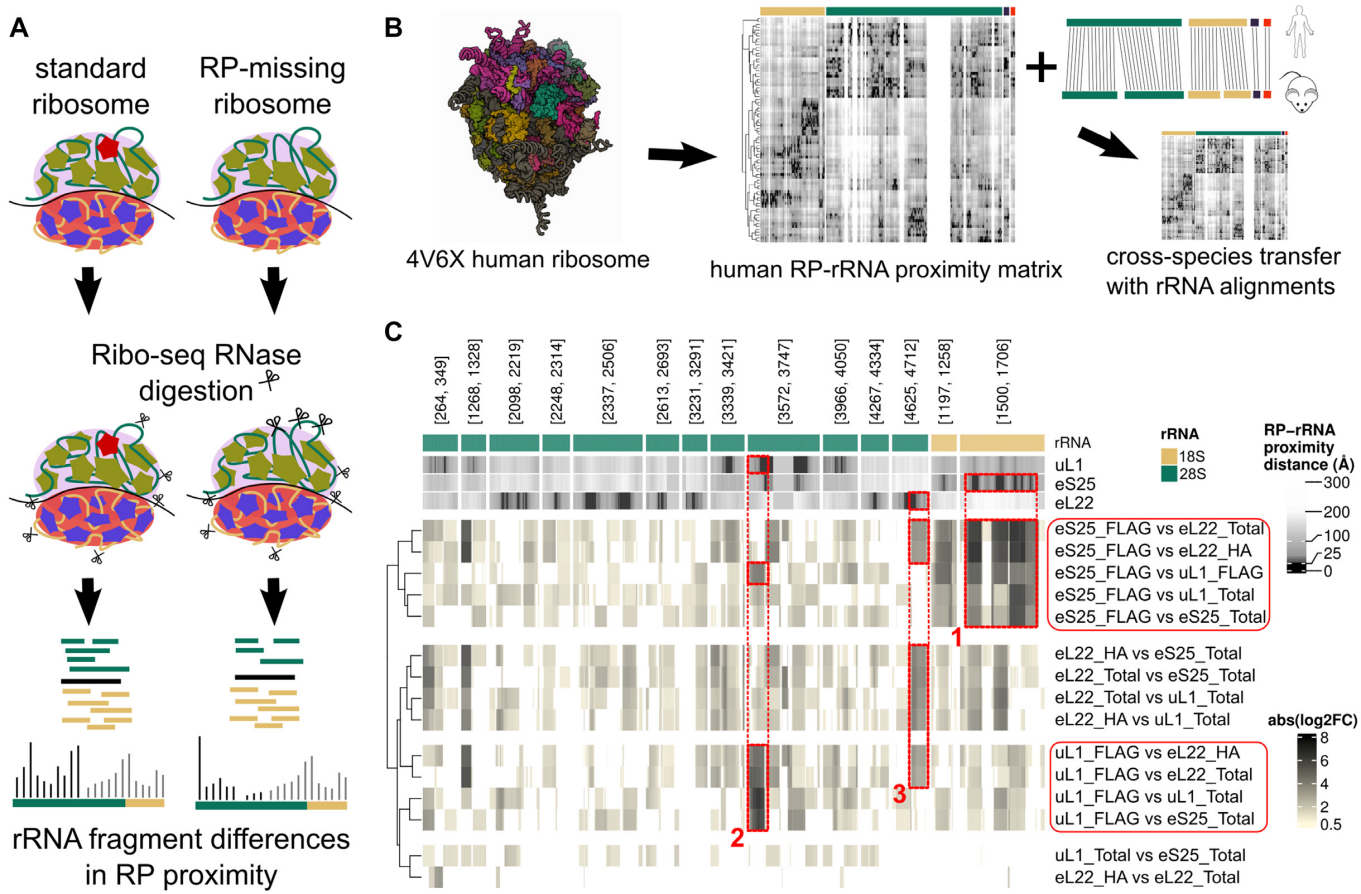
Tracking rRNA fragment changes to RP-mediated ribosome heterogeneity. (**A**) Our proposed model suggests that RP incorporation difference creates changes in Ribo-seq produced rRNA fragments specifically at rRNA positions in RP’s vicinity. (**B**) To be able to track rRNA fragments to RP and rRNA positions, 3D structure of the ribosome is transformed into a RP–rRNA proximity matrix where rows are RPs, columns are rRNA positions and values are RP–rRNA distances in Å. In this visualisation, darker colors represent the closest positions. (**C**) Changes in rRNA fragment abundance are most prominent at rRNA positions in close vicinity to RPs that change incorporation between compared populations. In this heatmap, top row indicates the rRNAs, following three rows indicate RPs distance to given rRNA positions, and the last 15 rows show the rRNA fragment change at given rRNA positions for given comparisons. Note that, this change is colored based on the absolute logFC levels where positions with insignificant changes (adj *P* > 0.05 or abs(log_2_FC) < 0.5) are given in white.

**Figure 3.**
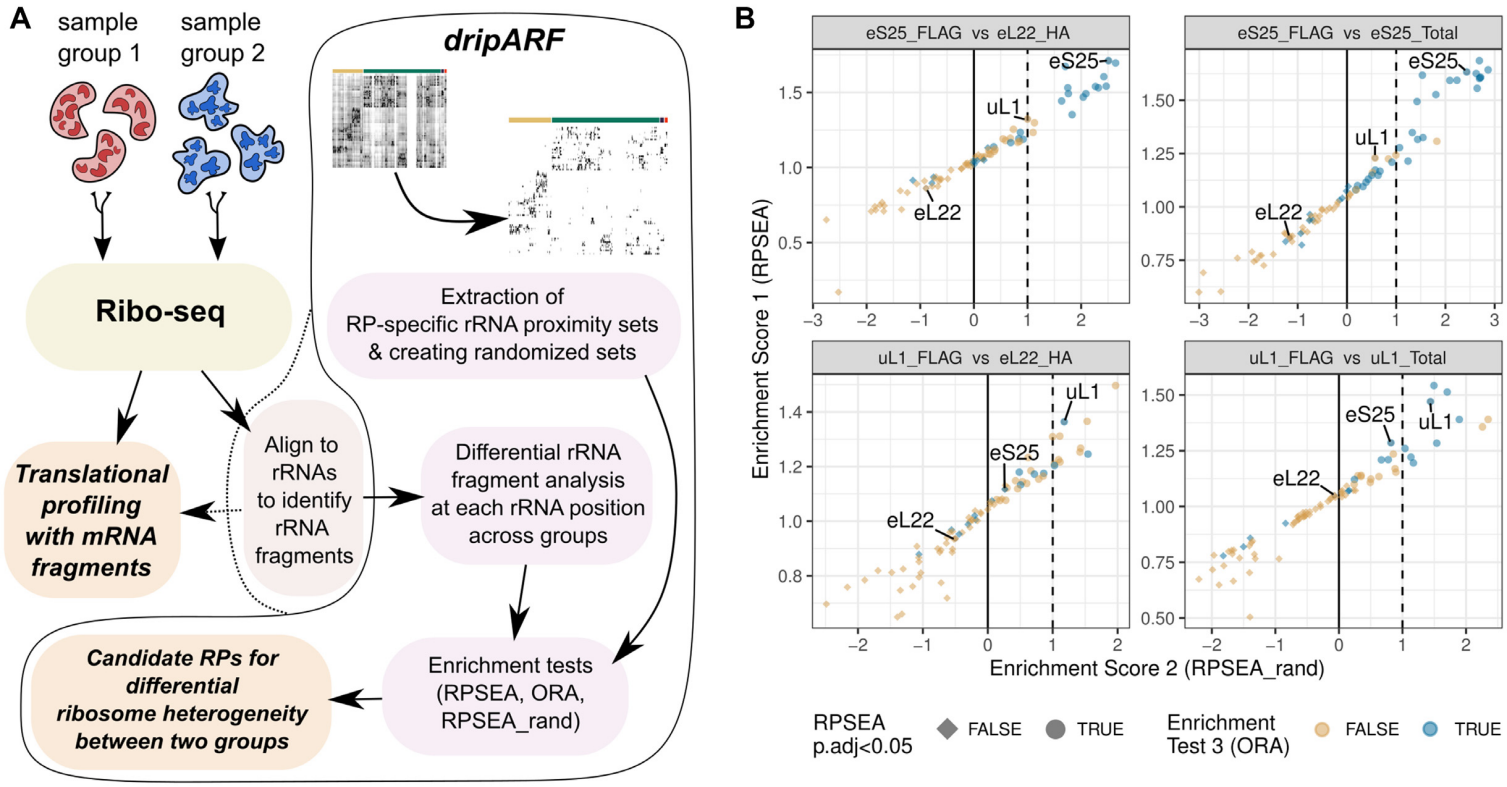
The dripARF method and benchmark results. (**A**) The schema for the dripARF pipeline. After preprocessing, Ribo-seq reads are mapped to rRNAs by the user, which is usually a standard step before translational profiling with mRNA fragments. This rRNA alignments are fed into dripARF pipeline for differential positional rRNA abundance analysis, which is followed by enrichment tests for RP–rRNA contact sets, leading to predictions for which RPs give rise to ribosome heterogeneity between compared sample groups. (**B**) Benchmark results with the Ribo-seq data from different ribosome populations (2). Each panel corresponds to a specific comparison that is titled on top. Axes, colors and shapes correspond to enrichment test results (described in Materials and Methods) performed by the dripARF pipeline. Top predictions are the ones closest to the top-right corner and expected to have a blue color and circle shape. RPs of interest are highlighted.

**Figure 4.**
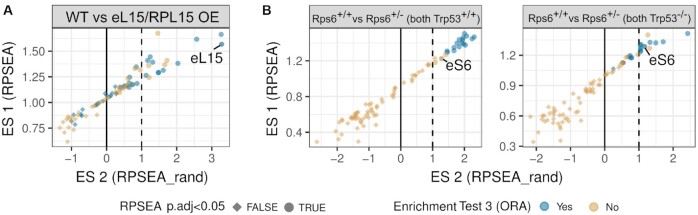
The dripARF method and ribosome heterogeneity predictions in (**A**) eL15/RPL15 overexpression (23) and (**B**) eS6/RPS6 heterozygosity (22) datasets. Each panel shows a dripARF prediction in a different comparison, where axes, colors and shapes correspond to dripARF enrichment test results. Top predictions are the ones closest to the top-right corner and expected to have a blue color and circle shape. RPs of interest are highlighted.

**Figure 5.**
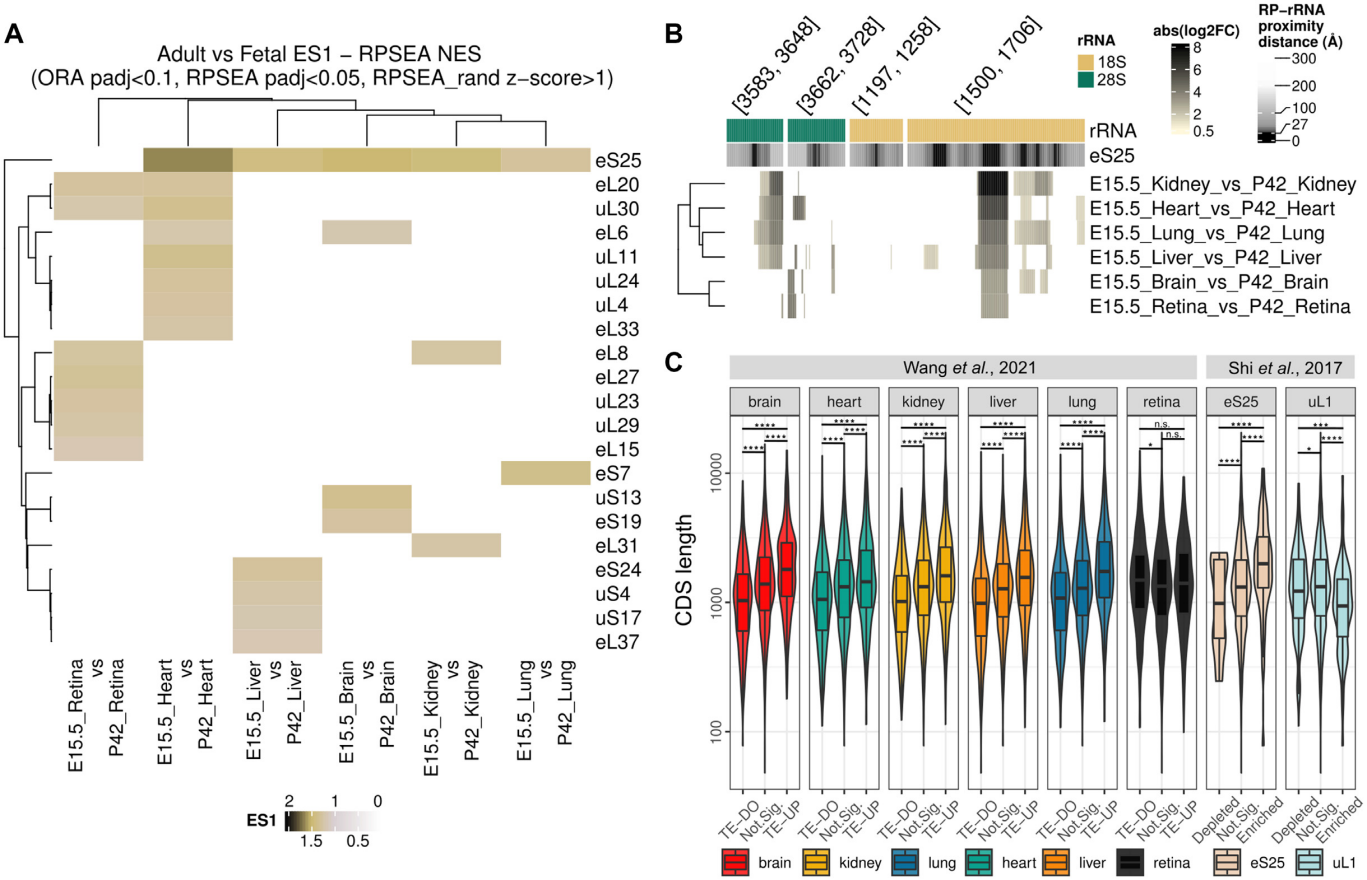
eS25/RPS25 is heterogeneous between fetal and adult ribosomes in mice, with a potential consequence on selective mRNA translation. (**A**) Enrichment score 1 (RPSEA) heatmap for different RPs (rows) in dripARF ribosome heterogeneity predictions in selected comparisons (columns). Predictions are based on Ribo-seq data (20), generated with the adult and fetal stage of 6 organs in mice; heart, retina, liver, kidney, lung and brain. (**B**) Closer look at rRNA fragment abundance change at rRNA positions in the eS25/RPS25 vicinity shows consistency across fetal-vs-adult comparisons. In this figure, the layout is the same as Figure 2C. (**C**) Translation efficiency changes in adult-vs-fetal comparisons, and mRNA association with eS25-enriched ribosomes is significantly associated with mRNA CDS length. In the figure, CDS length (y-axis) distribution of genes are given separately for each comparison, where genes are separated on the x-axis, into different TE groups for the first dataset and into association groups with specific ribosome populations for the second dataset.

All public datasets were downloaded through the NCBI SRA platform with the dataset-specific accession ids. For the analyses performed in Figures 1 and 5, raw Ribo-seq data for adult and fetal mouse organs were accessed using the accession id SRP100063 (20). For the post processing of raw reads, adapter sequence *AGATCGGAAGAGCACACGTCTGAACTCCAGTCA* was removed using the cutadapt tool (21) with following parameter settings; *-match-read-wildcards -m 15 --discard-untrimmed --action=trim --error-rate=0.15*. For the benchmark analyses with different ribosome populations in Figures 1–3, raw Ribo-seq data was accessed using the SRP064202 accession ID (2). For the additional benchmark data shown in Figure 4, eS6/RPS6-based ribosomopathy and eL15/RPL15 overexpression datasets were accessed using the SRP218125 (22) and SRP241899 (23) accession IDs. Adapter sequences, *CTGTAGGCACCATCAAT* and *TGGAATTCTCGGGTGCCAAGG* of these datasets were removed from raw reads as described above. Trimmed reads of all datasets were mapped to aforementioned rRNA sequences using the TopHat aligner (16).

### Translation efficiency analysis

Differential translation efficiency analysis in mouse adult and fetal organs (20) was performed with RiboDiff (24). Its input consisted of transcript quantifications of primary transcripts, decided based on APPRIS annotation, excluding the genes with low sequencing depth. Transcript quantification of RNAseq and RiboSeq reads were done with Salmon (25) for which protein-coding transcript sequences were obtained from gencode vM21 annotation. Note that, for Ribo-seq transcript quantifications, UTRs and duplicated sequences were removed prior to the run.

## RESULTS

### Ribo-seq produced rRNA fragments differ across samples and ribosome populations

Since its development, Ribo-seq has revolutionized the study of mRNA translation. However, as the protocol calls for the enrichment of ribosomes, high levels of rRNAs are a major problem with the technique, and they are routinely minimized using both experimental and bioinformatic depletion methods. We have recently published an approach to optimize rRNA depletion, which relies on the identification of the most common rRNA fragments in the data, and the design of oligos to specifically deplete them (12). During the development of this approach, we noticed patterns of rRNA fragments in the data that were highly reproducible in replicates, but differed across groups. While there were several protocol-related explanations for such variability (choice of RNase for example), one attractive explanation was that fundamental differences in the ribosome resulted in different digestion patterns. It is known that the RP complement of the ribosome is variable, and it is possible that differences in RPs cause the observed patterns. To confirm our observation of reproducible rRNA fragment patterns, we used a dataset whereby Ribo-seq was performed in multiple mouse organs at E15.5 (26). PCA analysis of the rRNA fragments produced by Ribo-seq showed that although replicates largely clustered together, different organs clustered separately, suggesting fundamental differences in how the rRNA was degraded in different tissues during the protocol (Figure 1A). While this supported our hypothesis, many other variables could still explain the observed differences, such as differences in endogenous RNase levels for example (27).

To exclude this possibility and to question whether RP heterogeneity could give rise to such differences in rRNA digestion, we next analyzed a dataset in which different ribosomal populations were isolated from the same cells (2). In that study the authors tagged uL1/RPL10A, eS25/RPS25 and eL22/RPL22, and used immunoprecipitation to isolate ribosomes containing those specific RPs, as well as sucrose gradient approach to isolate all ribosomes of the cell (Figure 1B). This allowed us to study rRNA digestion differences caused by differences in ribosome populations, independently of other potential variables such as endogenous RNases and RNase inhibitors. As can be seen in Figure 1C, the different populations still cluster separately in PCA analysis, suggesting that ribosomes with different RP complements have different rRNA digestion patterns. This is also supported by the rRNA-fragment based sample similarities that is calculated separately for every sample pair (Supplementary Figure S1). Further analysis and visual inspection of the rRNA fragment abundances revealed that there were a few comparison-specific hotspots with the most prominent rRNA fragment differences, which are consistent across replicates (Figure 1D). This suggests that the different RP complements of the ribosome populations may be directly causing the differential rRNA digestion.

### rRNA fragment differences can be traced back to rRNA-RP contact points

In order to understand the origin of the rRNA fragment differences that we observed, we hypothesized that the presence or absence of an RP would change the digestion patterns of rRNA at the residues that are in close proximity, through direct protection from RNase (Figure 2A).

We therefore analyzed the published structure of the human ribosome (14), and created an RP–rRNA proximity matrix (see Methods). This provided us with the distance of every rRNA position from every RP, allowing us to map RP-specific RP–rRNA contact points, which we defined as any rRNA position within ∼27.4 Å of the alpha carbon atom of the closest amino acid, as measured from the sugar backbone of the rRNA. We could therefore characterize a set of RP–rRNA contact points for each RP, and compare the observed rRNA fragment differences to these sets (Figure 2A and B). As the rRNA fragment data was produced from mouse embryonic stem cells, the comparison was carried out with a mouse-specific RP–rRNA proximity matrix, generated using rRNA alignments between mouse and human (see Methods).

For visual inspection, we quantified the rRNA fragment change at each rRNA position between the ribosome populations from the data above. This data included Ribo-seq from 6 different ribosome populations: uL1/RPL10A-enriched ribosomes, eS25/RPS25-enriched ribosomes, and eL22/RPL22-enriched ribosomes, plus total ribosome controls from each. When the rRNA fragment changes between these samples were compared to the RP–rRNA proximity matrix, each was found to have a consistent and predictable pattern, especially around the rRNA positions that are close to the differentially enriched RPs in the compared ribosome populations. For example, ribosomes enriched for eS25/RPS25 showed the most prominent differential rRNA fragment abundance around the defined contact points for this RP (Figure 2C, box 1). A similar observation was made for uL1/RPL10A containing ribosomes (Figure 2C, box 2), however, no eL22/RPL22-specific rRNA fragment changes were detected. As eL22/RPL22 is considered invariant across all ribosome populations, no relative changes would be expected in this group, confirming the specificity of the approach. It is also worth noting that this approach could also detect a signal caused by the presence of the HA-tag on eL22/RPL22, which was observed in all samples expressing eL22/RPL22-HA, both following affinity purification, and in total ribosomes from these samples (box 3 in Figure 2C). Moreover, there was a relationship between the distance of an rRNA position from each of these RPs, and the likelihood that its abundance would be altered following the enrichment of the RP. Global analysis of the relationship between rRNA fragment abundance changes and the distance of these rRNA positions from the studied RPs supported this observation (see Supplementary Figure S2).

These results suggest that differential RP incorporation into the ribosome can be detected through the analysis of rRNA fragments in Ribo-seq data.

### Accurate prediction of differential ribosome heterogeneity with dripARF

In order to automate this analysis and enable prediction of differential ribosome heterogeneity across any samples, we created the dripARF pipeline. In dripARF, to apply statistical rigor we took an approach akin to differential gene expression, which we can then follow with over-representation analysis (ORA) and gene-set enrichment analysis (GSEA, RPSEA in dripARF). In this analogy, each ‘gene’ refers to a rRNA position, ‘expression’ refers to position-specific rRNA fragment abundance, and the ‘geneset’ corresponds to an RP contact set, which we have defined above. With RP contact sets consisting of all the rRNA contact points for a specific RP, we test if differential rRNA abundance is enriched in these RP-specific rRNA positions, which could then hint to incorporation change of certain RPs causing different rRNA digestion between samples. Alongside the RPSEA and ORA enrichment tests, we have also created dummy rRNA position sets to control for background rRNA fragment abundance variations, creating the RPSEA_rand enrichment score (see Materials and Methods). In the dripARF workflow (Figure 3A), after normalization of sequencing depth across samples, we quantify the rRNA abundance change at each rRNA position and using the statistical measures on position-specific differential rRNA abundance, we test for enrichment of certain RP contact sets. Overall, the method allows us to predict differential RP incorporation, between samples in any given Ribo-seq dataset.

We first tested dripARF on the Ribo-seq data described above, enriched for different ribosome populations. When comparing uL1/RPL10A pull-down to either the matched total ribosomes, or to the control eL22/RPL22 pull-down, uL1/RPL10A was among the top candidates predicted by dripARF, validating the accuracy of the tool (Figure 3B). This was also the case for eS25/RPS25, although it is worth noting that in this example dripARF also predicted several RPs in the vicinity of eS25/RPS25 (uS13/RPS18, uS7/RPS5 and uS9/RPS16), suggestive of a broader structural change in the region (Supplementary Figure S3). Reassuringly, eL22/RPL22 was not predicted in any comparison.

To test the pipeline on other independent datasets, we analyzed a dataset in which eL15/RPL15 was over-expressed (23). In this study the authors showed that over-expression of this RP enhanced metastatic growth or tumors in multiple organs via an altered translational program. Analysis of this data predicted eL15/RPL15 to be differentially incorporated following eL15/RPL15 over-expression (Figure 4A), further supporting the power of dripARF and highlighting its potential in the reanalysis of previously published data. Interestingly the pipeline also predicted several RPs that are in close proximity to eL15/RPL15, suggesting that the pipeline may be detecting structural changes around this RP following over-expression (Supplementary Figure S4).

Finally, we made use of a study in which eS6/RPS6 haploinsufficient embryos were subjected to Ribo-seq, alongside their wild type littermates, both in the presence and absence of p53 (22). This haploinsufficiency results in a 50% decrease in the level of eS6/RPS6 in these animals. We accessed the Ribo-seq data, and analyzed it using the dripARF pipeline, which predicted eS6/RPS6 as being heterogenous in all relevant comparisons (Figure 4B).

### eS25/RPS25 may contribute to ribosome heterogeneity between fetal and adult tissues

It has been suggested that ribosome heterogeneity may play a role in development, and several studies support this notion. Mutation of RPs are known to cause remarkably tissue-specific developmental abnormalities in ribosomopathies, including Diamond-Blackfan anemia and 5q-syndrome (28,29). Mutations in uL18/RPL5 cause cleft palate (30), eL24/RPL24 heterozygosity in mice results in a kinked tail and the formation of additional digits (31), and knock-down of each of 19 RPs in zebrafish result in gross development deformities (32). It has also been shown that eL38/RPL38 regulates the translation of Hox mRNAs and plays a vital role in skeletal patterning as a result (33). We therefore used the dripARF pipeline to assess whether RP incorporation into the ribosome changes during development. We used data from a study in which Ribo-seq was carried out in both pre- and post-natal mouse tissue (E15.5 versus P42) (20). Surprisingly, despite the different tissue origins (ectoderm: brain, retina; mesoderm: heart, kidney; and endoderm: liver, lung), in five of six tissues eS25/RPS25 is predicted to be changing between embryonic tissue and the adult tissue from the same organ, with the exception being retina (Figure 5A, B, Supplementary Figure S5). Crucially, when adult tissues are compared to each other (Supplementary Figure S5), eS25/RPS25 is not predicted to be heterogeneous, suggesting that this is unique to the development process. Interestingly, dripARF also predicted eS25 to be variable between fetal tissues (Supplementary Figure S5), suggesting that eS25 is variable in fetal ribosomes, but then becomes saturated in adult tissue.

In order to understand the potential functional relevance of eS25/RPS25 in ribosomes, we analyzed the features of mRNAs with altered translation efficiency (TE) in adult compared to fetal tissue. This was calculated by combining the Ribo-seq and RNA-seq data from the same dataset (see Materials and Methods) (20). This analysis revealed that when compared to mRNAs that do not have altered TE, mRNAs with increased TE in adult compared to fetal tissue tend to have a longer CDS, while those with a decreased TE tend to have a shorter CDS (Figure 5C). This was true across all organs, except retina, which does not show differential incorporation of eS25. As the panel of analyzed mRNAs differed across organs, this gives us confidence that the observed effect is real. Furthermore, when we carried out the same analysis on eS25-enriched ribosomes (2), we found the same pattern, confirming the association in an independent dataset. The same association was not observed in uL1-enriched ribosomes, supporting the notion that eS25 specifically alters ribosome dynamics (Figure 5C).

As eS25/RPS25 is also known to play a role in IRES-mediated initiation (34), we also analyzed the translation efficiency (TE) of mRNAs known to have a functionally validated IRES in mice, which we identified using the IRESbase database (35). This revealed that adult tissue tended to show increased TE for these mRNAs (Supplementary Figure S6). The relationship was relatively weak however, due to the low number of genes with validated IRES in mice. The observation does suggest a potential future research direction in understanding the functional consequences of differential eS25/RPS25 incorporation in ribosomes.

## DISCUSSION

The recognition that heterogenous ribosomes play an important role in RNA translation has been a significant one. Our view of the ribosome as a monolithic macromolecule has had to be overturned, and we now understand that many things can lead to specialized ribosomal function, including RP heterogeneity (2), RP post translational modification (36), and rRNA structure and modification (3,37). However, the study of heterogeneous ribosomes had been hampered by the lack of techniques available to study it.

We have developed dripARF, a method of detecting RP heterogeneity through the analysis of rRNA fragments generated in Ribo-seq. The tool predicts differential RP incorporation into the ribosome, adding a significant layer of information that can be gleaned from Ribo-seq data. While it does not directly measure RP level (unlike MS-based methods), it provides a new way of probing ribosome composition, and one that is much needed. MS of ribosomal proteins is difficult due to their small size and lack of tryptic peptides. There is also currently little motivation to carry out such an analysis, as we lack indicators of ribosome heterogeneity. dripARF addresses this problem because it is an addition to Ribo-seq, which is an experiment that is likely to be carried out if RNA translation is implicated in a phenotype. The addition of dripARF to Ribo-seq analysis should open the field of ribosome heterogeneity significantly, informing follow-up experiments, and in some cases allowing the identification of the mechanism of translational alterations. Given the wealth of published Ribo-seq datasets, the tool already has enormous potential to provide new insights into many biological processes, as we have shown through the analysis of organ development. Additionally, dripARF may allow us to more deeply understand which RPs can be heterogeneous, and what determines this ability. For example, three of the four RPs we have shown to be heterogeneous in this manuscript (eS25/RPS25, uL1/RPL10A and eS6/RPS6) are found on the periphery of the ribosome, and it is predicted that RPs with such localization are unlikely to play a role in ribosome stability (Supplementary Figure S7). Such insights will open up many new research lines, and should provide a measure of just how common RP incorporation heterogeneity is, and its importance in health and disease.

There are several clear limitations to the approach, some of which are unavoidable due to lack of data and variations in the Ribo-seq protocol, and others that may be overcome with additional development of the tool. Most important is that while we have shown that dripARF can correctly identify differential incorporation of some RPs, it cannot be assumed that it will work correctly for all RPs. As more data is generated we will be able to improve and optimize the approach, however, until then, this should be kept in mind. For instance, structurally unresolved parts of the ribosome, like flexible rRNA segments or flexible portions of RPs, are excluded from our distance-based approach as we cannot calculate any distance measures for them. This may result in incomplete RP–rRNA contact point sets.

At present, our definition of contact point set is also quite crude, being any amino acid within ∼27.4 Å of an rRNA position (to a limit of 360 closest rRNA position contact points). However, as more Ribo-seq data is generated with known RP alterations, this definition will be able to be refined and improved, increasing the confidence in the results.

Additionally, dripARF occasionally detects changes in RPs in close proximity to the variable RP. A good example of this is in eS25/RPS25, where uS13/RPS18, uS7/RPS5 and uS9/RPS16 are also predicted as differential (Supplementary Figure S3). This is also the case with eL15/RPL15, as we predict eL13/RPL13 to be differentially incorporated (Supplementary Figure S4), and these two RPs are known to directly contact each other in the ribosome. This may be overcome as we refine our contact point definition, but may also be a sign of larger structural changes caused by the presence or absence of a specific RP. It is important to note, however, that the contact point sets for different RPs are unique, suggesting that the predicted changes in local RPs are not a result of ill-defined contact point sets, but may point towards broader ribosomal alterations caused by ribosome heterogeneity. Indeed, any other alterations that cause a structural change to the ribosome, such as ribosome associated proteins (RAPs), could potentially interfere and/or be detected using dripARF, but this will only become clear with use. It is known, for example, that rRNA modifications are also heterogeneous across samples (38,39). If such heterogeneity alters ribosome structure or composition, then it may be detectable using this approach.

Another important caveat to highlight is that we have decided against providing a directionality to the predicted change in RP incorporation. As changes in digestion patterns could alter the rRNA fragment population in many ways, it is difficult to make definite predictions. For example, our approach is based on the idea that increased RP incorporation into the ribosome would protect specific rRNA regions from digestion. However, this protection may result in longer rRNA fragments, which would then be lost during the size selection step of the Ribo-seq protocol. This would result in a depletion of that fragment from the sequencing data, and a dripARF prediction of less incorporation rather than more. While additional optimization of the tool may overcome this issue, at present we are uncomfortable predicting the directionality of a change in data with so many uncontrollable variables.

Several limitations also come as a result of the Ribo-seq protocol. One such limitation is the rRNA fragment size (as is mentioned above), which is specifically selected to match the mRNA protection by the ribosome (somewhere between 19 and 35 nucleotides, depending on the study). Although dripARF can currently correctly predict differential RP incorporation in the situations we have tested, there is no guarantee that the changes in other RPs will also result in rRNA fragments of this size. Similarly, rRNA depletion approaches are commonly applied, and the data from the depleted regions may be lost to dripARF analysis. If the primary goal of a Ribo-seq experiment is to probe the RP content of the ribosome, then it is probably wise to avoid rRNA depletion steps, in order to maximize rRNA reads. However, rRNA depletion was applied to all the data analyzed in the development and testing of dripARF, and despite this we have been successful in identifying the expected RP heterogeneity in all of these cases, clearly showing that this is not a necessity for its detection.

A more worrying confounding factor is in other drivers of differential rRNA digestion. It is known that both the endogenous RNases, and those used as part of the Ribo-seq protocol can affect rRNA digestion pattern (12). We recommend that dripARF is only applied to data from specific experiments, and not across experiments, to minimize variation introduced by protocol-to-protocol differences. Variations in rRNA digestion caused by endogenous RNases are a more difficult problem, and at present this is not corrected for in the dripARF pipeline. It is therefore prudent to confirm dripARF predictions using direct measurement of RP association with the ribosome. Finally, it is also possible that digestion artifacts may arise from alternative rRNA transcript usage, however, due to the high level of identity between alternative transcripts, we believe this to be minimal.

Although it is clear that RP heterogeneity exists in the ribosome, and can exert a significant regulatory role, it is currently unknown how important this function of ribosomes is. There is a great need for additional techniques to detect such heterogeneity, and dripARF fulfills this. We have shown that dripARF accurately detects heterogeneous ribosomes, and that it can be applied to datasets to provide novel information and hypotheses. Ultimately, we believe that dripARF will contribute significantly to the study of RP heterogeneity in the ribosome, and make the field more accessible to other researchers.

## Supplementary Material

gkac484_Supplemental_File

## Notes

Present address: Santiago Hernández-Pérez, NanoString Technologies Inc., Amsterdam, The Netherlands.
