# Supplementary material for: Identifying ribosome heterogeneity using ribosome profiling
Source: Nucleic Acids Res. 2022 Jun 10;50(16):e95. doi: 10.1093/nar/gkac484 (PMC9458444; doi:10.1093/nar/gkac484)
Supplement: gkac484_Supplemental_File [file gkac484_supplemental_file.pdf]

Identifying ribosome heterogeneity using ribosome profiling
(Supplementary Document)

Ferhat Alkan [1,*], Oscar G. Wilkins [2,3], Santiago Hernández-Pérez [1,†], Sofia Ramalho [1],

Joana Silva [1], Jernej Ule [2,3,4], William J. Faller [1,‡]

[1]Division of Oncogenomics, The Netherlands Cancer Institute, Amsterdam, The Netherlands
[2]The Francis Crick Institute, London, UK
[3]UCL Queen Square Motor Neuron Disease Centre, Department of Neuromuscular Diseases, UCL Queen Square Institute of Neurology, UCL, London, UK
[4]UK Dementia Research Institute Centre, King's College London, London, UK

List of Figures

Supplementary Figure 1 2
Supplementary Figure 2 3
Supplementary Figure 3 4
Supplementary Figure 4 5
Supplementary Figure 5 6
Supplementary Figure 6 7
Supplementary Figure 7 7

*Software-related correspondence should be addressed to f.alkan@nki.nl
†Present address: NanoString Technologies Inc., Amsterdam, The Netherlands
‡Corresponding Author, email: w.faller@nki.nl

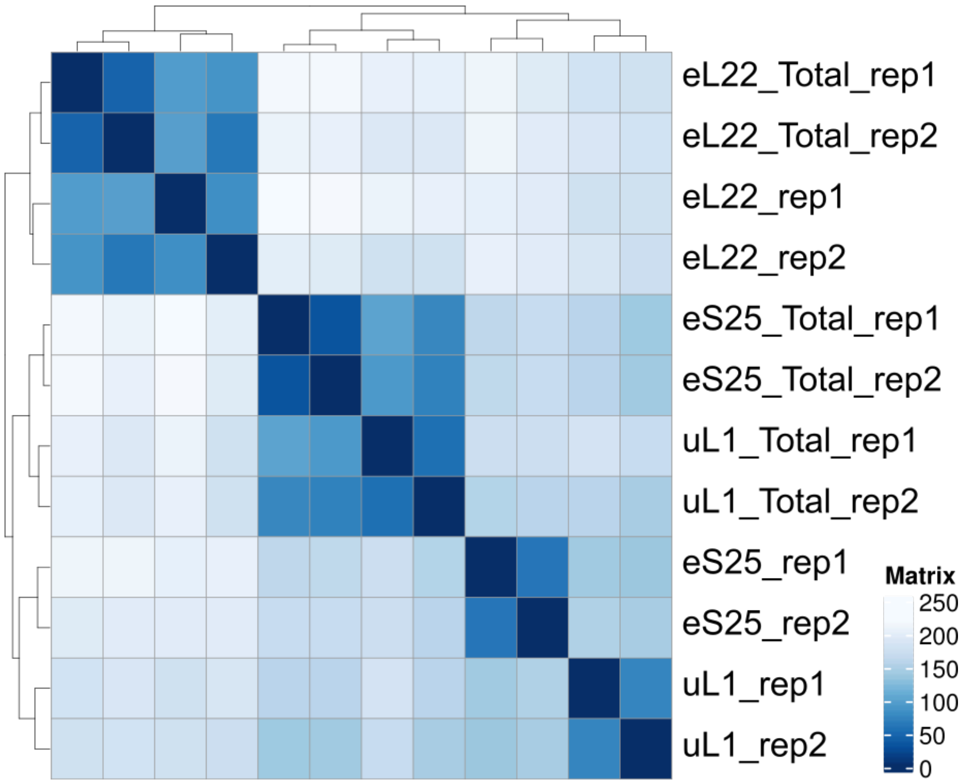

Supplementary Figure 1: Sample similarity distance matrix (hierarchical clustered) created with positional rRNA abundance measures for the Ribo-seq dataset with heterogeneous ribosome populations analyzed in Fig 1b-d, 2c & 3b.

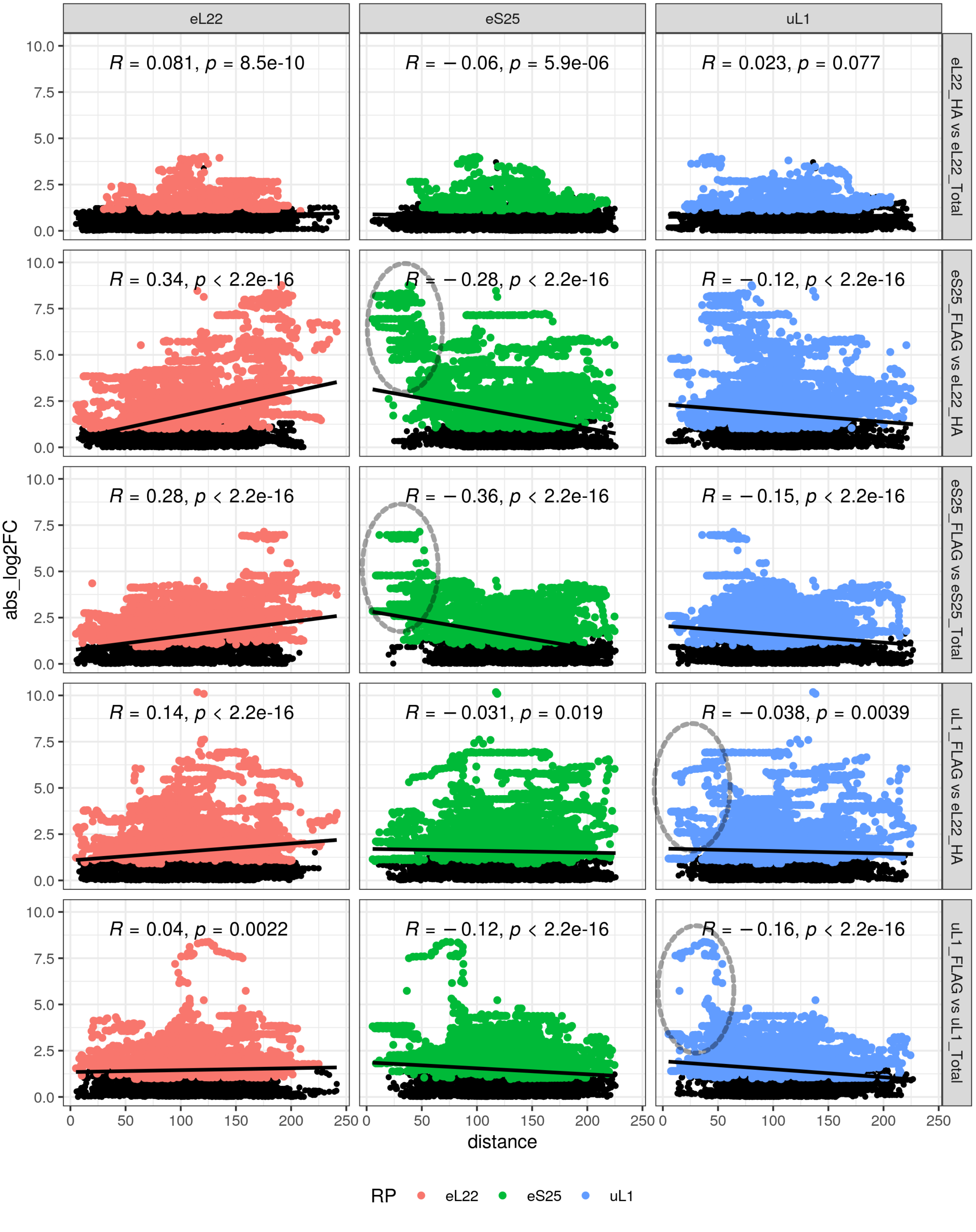

Supplementary Figure 2: RP-specific RP-rRNA distance vs comparison-specific rRNA abundance change scatter plots with Ribo-seq data from different ribosome populations. Each point represents a rRNA position, y-axis shows the rRNA fragment abundance change at that position and x-axis shows the distance to selected RP (eL22, eS25, eL1). Black color represent the rRNA positions that are not changing significantly and expected rRNA abundance changes in RP-proximal regions are highlighted in a dashed ellipse.

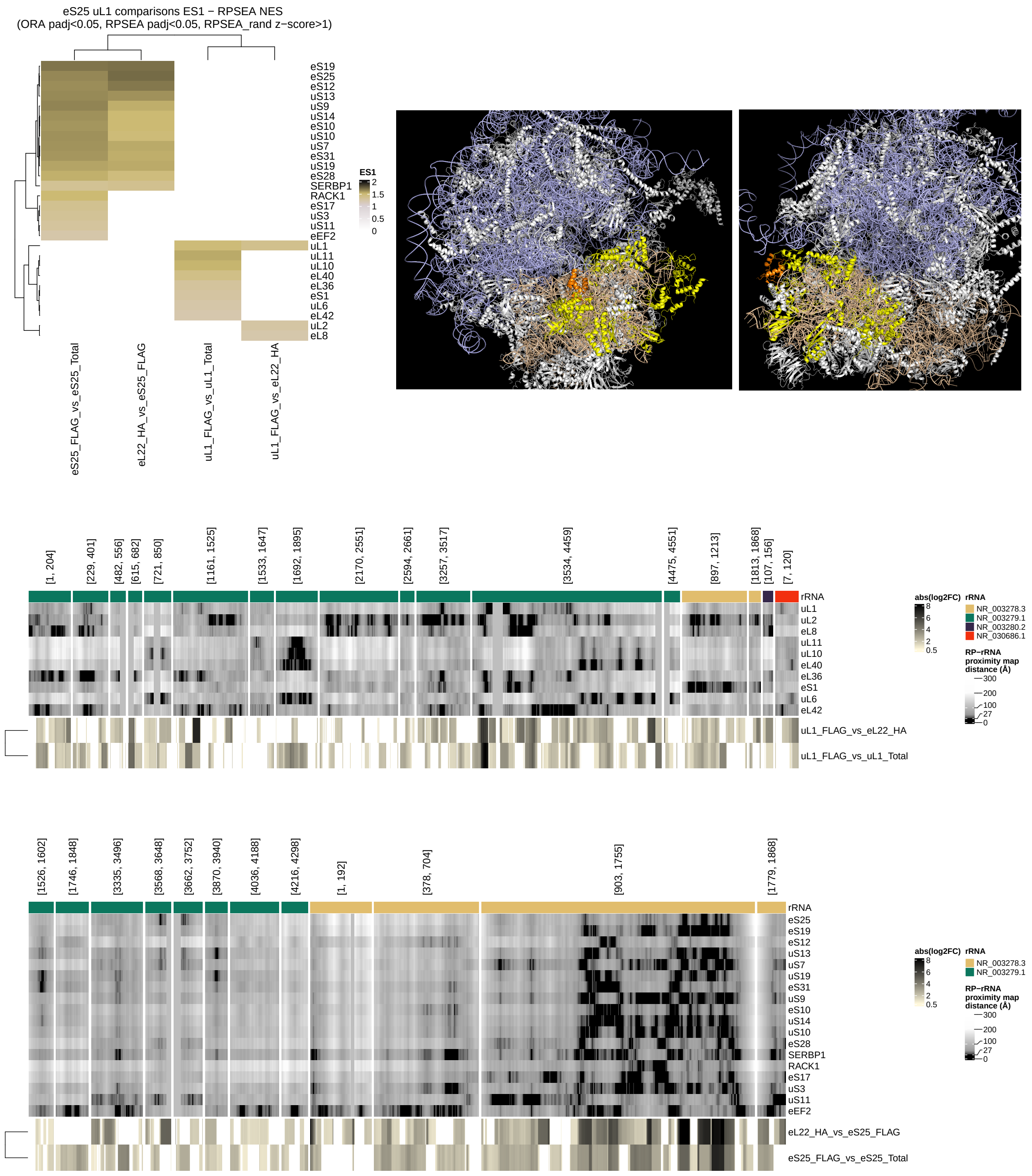

Supplementary Figure 3: Top-left: dripARF ribosome heterogeneity prediction results between selected populations that are given in x-axis. Heatmap color corresponds to Enrichment Score 1 of RPs given in the y-axis, where insignificant results are filtered out. **Top-right:** For eS25_FLAG comparisons, location of predicted RP overlaps (yellow) are shown within the 3D ribosome. Orange color shows the eS25/RPS25, light blue corresponds to the 28S rRNA, and wheat color shows the 18S rRNA. **Bottom:** Two heatmaps, generated seperately for eS25 and uL1 comparisons, show the location-specific rRNA abundance change and their overlap with the RP-rRNA proximity map. Layout is same as Fig. 2c in the original paper.

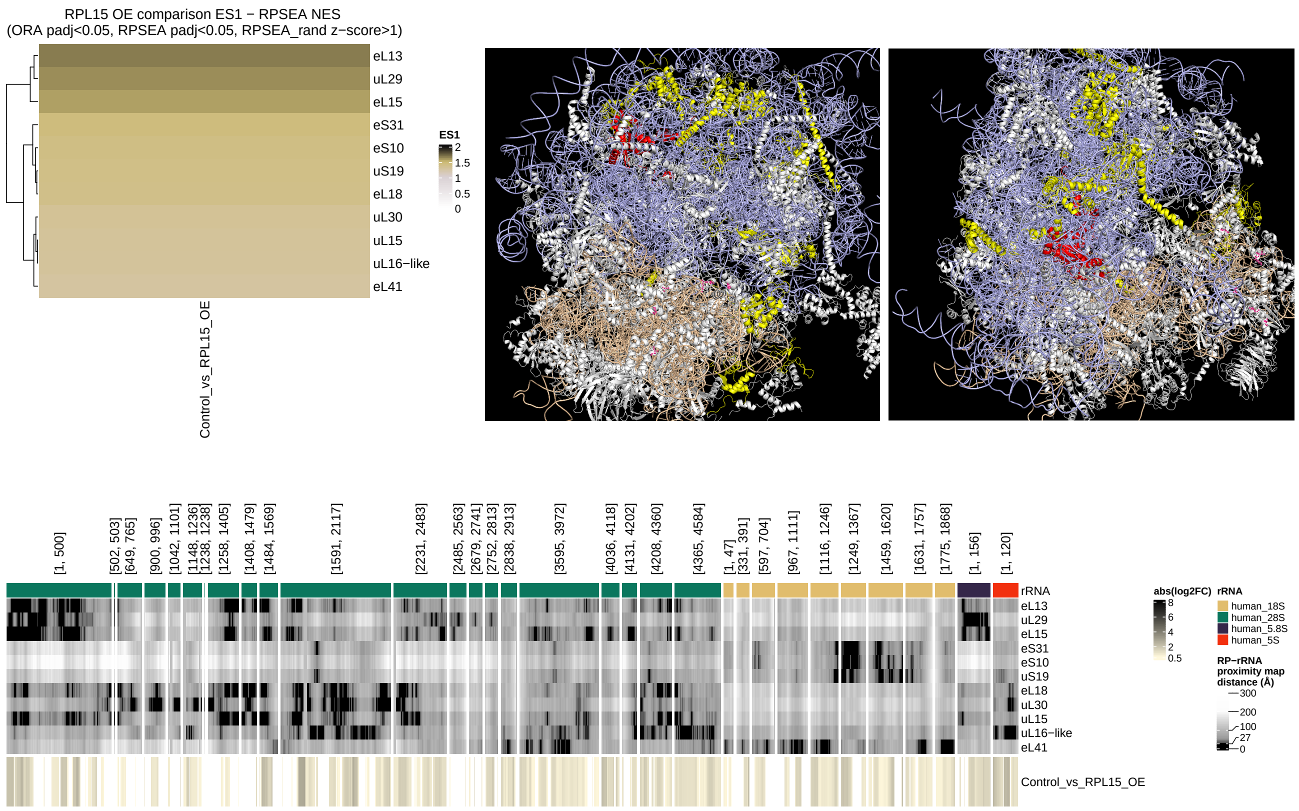

Supplementary Figure 4: Top-left: dripARF ribosome heterogeneity prediction results between eL15/RPL15 overexpression and WT samples. Heatmap color corresponds to Enrichment Score 1. **Top-right:** Ribosomal location of of the predicted RPs are highlighted with yellow within the 3D ribosome, whereas eL15/RPL15 is shown in red, light blue corresponds to the 28S rRNA, and wheat color shows the 18S rRNA. **Bottom:** Location-specific rRNA abundance change and their overlap with the RP-rRNA proximity map. Layout is same as Fig. 2c in the original paper.

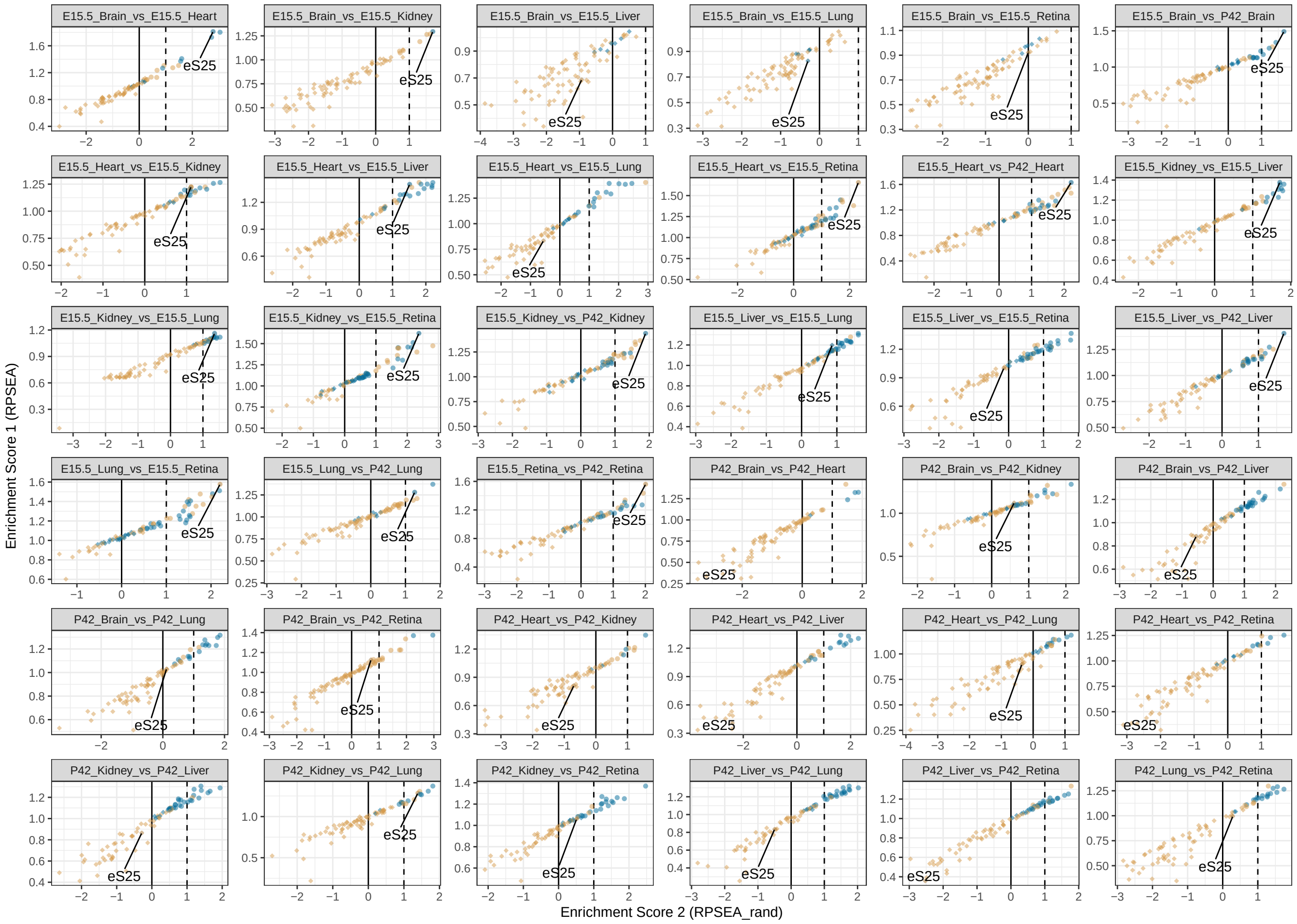

Supplementary Figure 5: dripARF ribosome heterogeneity prediction results for all fetal and adult tissue comparisons. One can observe that eS25/Rps25 is consistently predicted between adult and fetal stages of organs and across fetal tissue comparisons, however, not predicted in adult tissue comparisons.

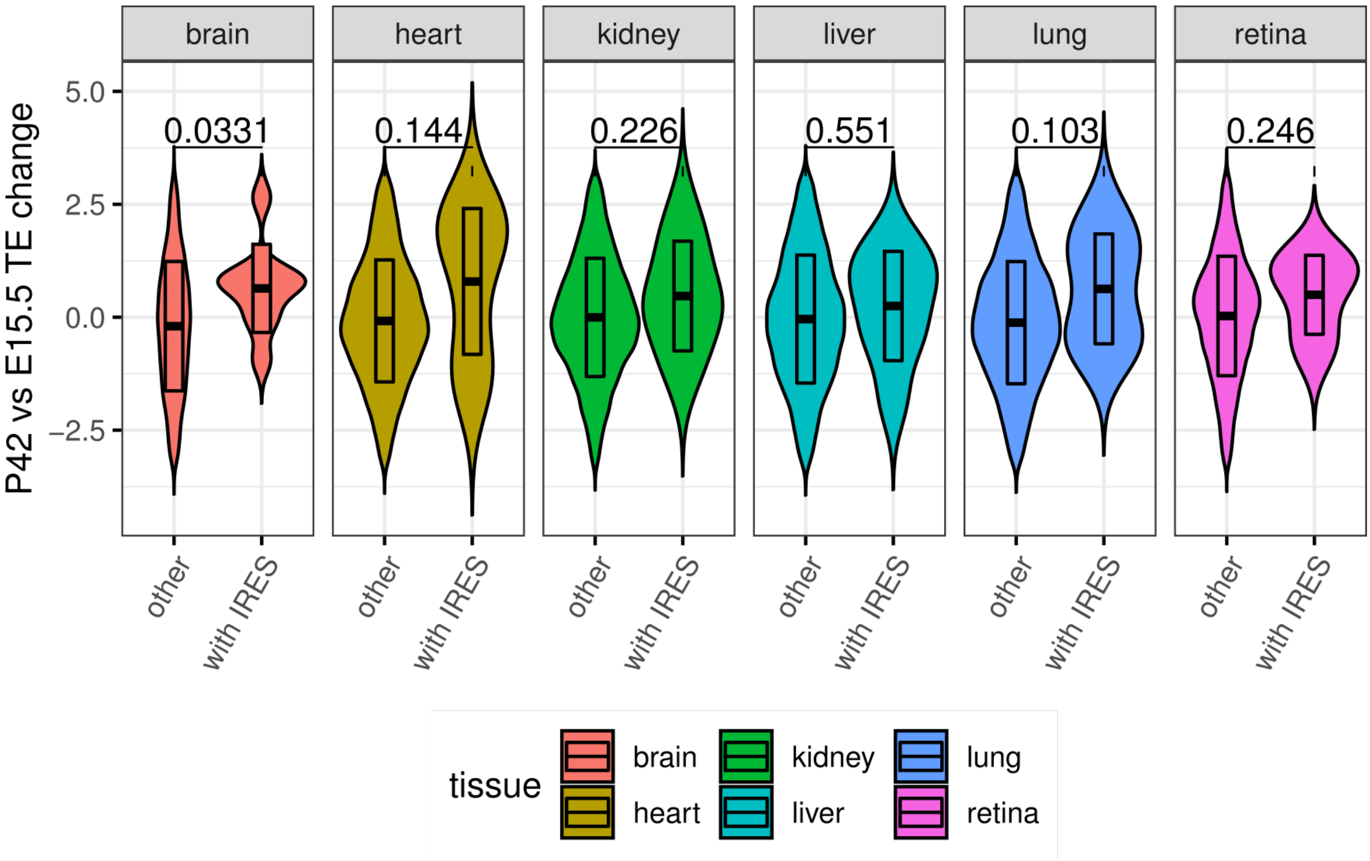

Supplementary Figure 6: TE change (y-axis) distribution of genes in adult-vs-fetal comparisons where genes are grouped on the x-axis based on whether they contain an IRES element or not. P-values are given on top when two distributions are compared.

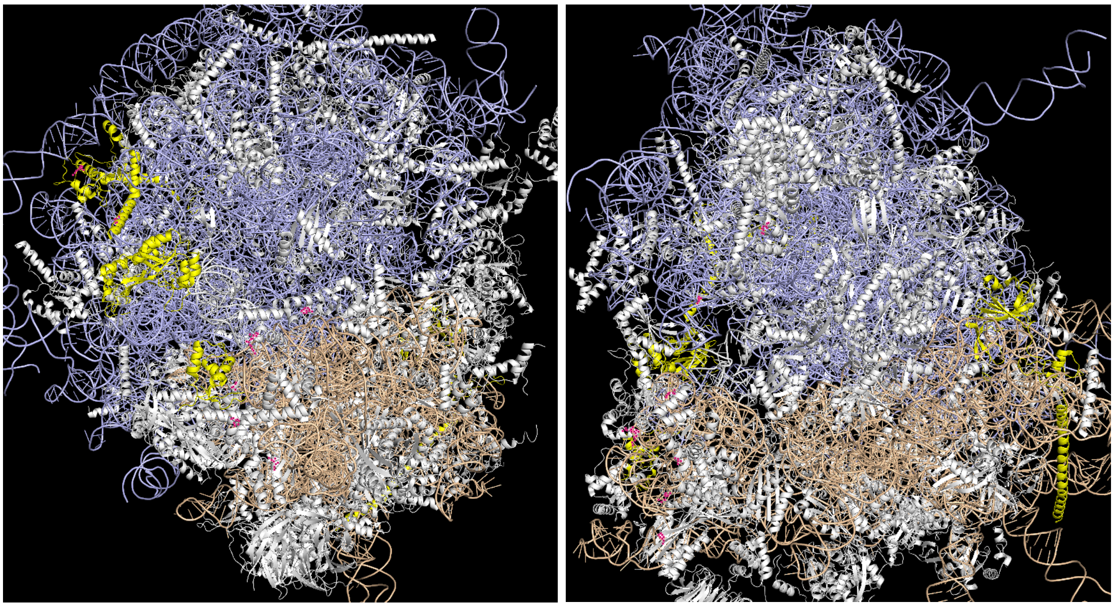

Supplementary Figure 7: We show that eS25/RPS25, eL1/RPL10A, eL13/RPL13 and eS6/RPS6 (all in yellow) are found on the periphery of the ribosome.
